# Impact of Somatic Conditions and Lifestyle Behaviours on Depressive Symptoms and Low Life Satisfaction among Middle-Aged and Older Adult Men in South Africa

**DOI:** 10.31083/j.jomh1809194

**Published:** 2022-09-21

**Authors:** Karl Peltzer, Supa Pengpid

**Affiliations:** 1 Department of Psychology, University of the Free State, 9300 Bloemfontein, South Africa; 2 Department of Psychology, College of Medical and Health Sciences, Asia University, Wufeng, 41354 Taichung, Taiwan; 3 Department of Health Education and Behavioral Sciences, Faculty of Public Health Mahidol University, 10400 Bangkok, Thailand; 4 Department of Research Administration and Development, University of Limpopo, 0727 Polokwane, South Africa

**Keywords:** chronic diseases, incident depression, persistent depression, low life satisfaction, longitudinal study, South Africa

## Abstract

**Objective:**

The purpose of this study was to assess the association between somatic disorders, lifestyle factors, incident and persistent depressive symptoms, and low life satisfaction in a longitudinal study in South Africa.

**Methods:**

We analyzed longitudinal data from two consecutive waves, 2346 men aged 40 years or older in 2014/2015 in wave 1 and 1864 men of wave 1 in 2018/2019 in wave 2 of the “Health and Ageing in Africa: A Longitudinal Study of an International Network for the Demographic Evaluation of Populations and their Health (INDEPTH) Community in South Africa (HAALSI)”.

**Results:**

In total, 360 of 1932 male participants without depressive symptoms in wave 1 (24.3%) had incident depressive symptoms in wave 2 and 77 of 349 men had depressive symptoms in both waves 1 and 2 (persistent depressive symptoms). In all, 457 of 1258 male participants without low life satisfaction in Wave 1 (47.6%) had incident low life satisfaction in Wave 2, and 360 of 998 men had low life satisfaction at both Wave 1 and 2 (persistent low life satisfaction). In the unadjusted logistic regression analysis, having kidney disease and living with HIV had greater odds of incident depressive symptoms. In adjusted analysis, alcohol dependence (Adjusted Odds Ratio-AOR: 4.54, 95% Confidence Interval-CI: 1.05–19.66) was positively correlated and 1–7 and 8–11 years of education (AOR: 0.45, 95% CI: 0.27–0.74, and AOR: 0.20, 95% CI: 0.07–0.54, respectively) were negatively associated with persistent depressive symptoms. Increasing age increased the odds (AOR: 1.03, 95% CI: 1.01–1.04), while higher education (≥12 years) (AOR: 0.50, 95% CI: 0.33–0.76), and high physical activity (AOR: 0.68, 95% CI: 0.52–0.89) decreased the odds of incident low life satisfaction. Increasing age (AOR: 1.03, 95% CI: 1.02–1.04) and tobacco use (AOR: 1.64, 95% CI: 1.23–2.19) increased the odds and high physical activity (AOR: 0.73, 95% CI: 0.56–0.96) decreased the odds of persistent low life satisfaction.

**Conclusions:**

Of the seven chronic conditions and five lifestyle factors evaluated, alcohol dependence increased the odds of persistent depressive symptoms and low physical activity, and tobacco use increased the odds of incident and/or persistent low life satisfaction among men in rural South Africa.

## 1. Introduction

There has been a demographic and epidemiological transition that has increased ageing and chronic noncommunicable diseases, including in lower-resourced countries [1]. In a multi-country cross-sectional study, the number of somatic conditions increased with age, while co-morbidity of depression with somatic disorders decreased with age [2]. Lifestyle factors, such as tobacco use, heavy alcohol use, inadequate fruit/vegetable intake, and physical inactivity, have traditionally been linked with the development of non-communicable diseases [3]. However, more recent studies show a positive association between health risk behaviors and poor mental health among both men and women [4,5]. In South Africa, poor mental health has been shown to have negative socioeconomic impacts [6]. Few longitudinal studies investigated the relationship between having somatic disorders, lifestyle factors and depression and life satisfaction among middle-aged and older men, in particular in Africa.

Generally, among men and women, in a longitudinal study among middle-aged and older adults in China, specific self-reported diseases, including stomach/other digestive diseases, diabetes, arthritis/rheumatism, and kidney diseases, were associated with incident depression [7]. Other studies also found an association between heart disease and incident depression among both sexes [8,9]. Kidney disease and dyslipidemia were associated with low life satisfaction among men and women in China [10].

Regarding lifestyle factors, in systematic reviews of prospective studies, Schuch *et al*. [11] conclude that physical activity can protect against depression, and Dishman *et al*. [12] found that physical activity is inversely associated with incident depression. In a prospective study in four countries, Cabello *et al*. [13] found that among the different health risk behaviors assessed, tobacco use was associated with incidence depression without gender differences, and those who engage in heavy drinking between both sexes can become more likely to become depressed over time [14]. In a further systematic review of prospective studies among middle-aged and older adults, higher consumption of fruit and vegetables was associated with a lower odds of incident depression [15]. In a study among middle-aged and older adults, higher physical activity was associated with greater life satisfaction [16], and in a longitudinal study, higher physical activity increased the odds of psychological well-being [17]. Based on these studies reviewed it is hypothesized that somatic disorders may negatively impact on mental health and healthy behaviors may positively impact on mental health. It is theorized that complex interactions exist between determinants, such as stress from somatic disorders, behaviors and mental health and well-being [18].

It is unclear if somatic disorders, including human immunodeficiency virus (HIV), and lifestyle factors are associated with incident and/or persistent depressive symptoms and low life satisfaction among men in Africa, which prompted this study among men in South Africa.

## 2. Methods

### 2.1 Sample and Procedure

We analyzed longitudinal data from two waves of the “Health and Ageing in Africa: A Longitudinal Study of an INDEPTH Community in South Africa (HAALSI)”. Detailed information on the sampling strategy has been previously described [19]. Briefly, participants were randomly sampled from the “Agincourt health and sociodemographic surveillance system (AHDSS)”, in South Africa. The first survey (wave-W1) was conducted between November 2014 to November 2015, with a sample of 2346 men aged 40 years or older (response rate 85.9%) [19], and the second survey (W2) between October 2018 and November 2019 among 1862 men of the Wave 1 HAALSI cohort (338 died over the follow-up: 14.4%, 127 declined participation: 5.4%, 19 were not found: 0.8%), and response rate: 94%. Data was collected by trained field workers using computer-assisted personal interviewing (CAPI) at the homes of participants.

### 2.2 Measures

#### 2.2.1 Outcome Variables

At baseline and follow-up, depressive symptoms were evaluated with the “Center for Epidemiological Studies Depression Scale eight-item scale (CES-D 8)” [20] or CES-D 20 modified to CES-D 8 [21], with “a cutoff of three or more symptoms that signify depressive symptoms” (Cronbach’s alpha 0.7 at baseline and 0.8 at follow-up).

At baseline and follow-up, life satisfaction was sourced from the item, “All things considered, how satisfied are you with your life as a whole these days? Use a 0 to 10 scale, where 0 is dissatisfied and 10 is satisfied” [19]. Low life satisfaction was defined as 0–6 (=below the median 7) and high life satisfaction as 7–10.

#### 2.2.2 Somatic Disorders

Hypertension was assessed based on the last two of three blood pressure measurements and defined: “if systolic blood pressure was greater than or equal to 140 mmHg or diastolic blood pressure was 90 mmHg or higher, or if the use of antihypertensive medication was reported at the time of the the interview” [19].

Dyslipidemia was defined as: “total cholesterol >6.21 mmol/L, high-density lipoprotein-cholesterol (HDL-C) <1.19 mmol/L, low-density lipoprotein-cholesterol (LDL-C) >4.1 mmol/L, triglycerides >2.25 mmol/L; reported ever diagnosed with high cholesterol; or if medication use is reported at the time of interview” [19].

Diabetes was “classified with fasting glucose (defined as >8 hours) >7 mmol/L (126 mg/dL) or non-fasting glucose level >11.0 mmol/L (200 mg/dL); reported ever being diagnosed with diabetes; or if use of medication is reported at the time of interview” [19].

Anemia was defined as ‘a blood hemoglobin concentration of <13g/dL for men or <12g/dL for women’ [22].

Stroke, heart attack, angina, and/or heart failure (cardiovascular disease), kidney disease, and HIV status were assessed by self-reported diagnosis [19].

#### 2.2.3 Lifestyle Factors

Current tobacco use was measured with questions on current tobacco smoking and current smokeless tobacco use [19].

Alcohol dependence was measured using the CAGE questionnaire [23]; Cronbach’s alpha was 0.8 in the present study.

Fruit and vegetable intake was sourced from two items, “How many servings of fruit/vegetables do you eat on a typical day? (on any one day)” (examples and serving sizes were demonstrated with show-cards) [19].

Physical activity was measured and classified with the “General Physical Activity Questionnaire (GPAQ)” [24, 25].

Body mass index (BMI) was measured and classified into “underweight (<18.5 kg/m^2^), normal weight (18.5–24.9 kg/m^2^), overweight (25–29.9 kg/m^2^), and obesity (≥30 kg/m^2^)” [19,26].

Sociodemographic data assessed consisted of marital, and asset-based household wealth status, country of birth, years of education, age and sex [19].

### 2.3 Data Analysis

Descriptive statistics are used to report on the proportion of people with incident and persistent low life satisfaction and depressive symptoms. The first logistic regression model excluded those with depressive symptoms or low life satisfaction at baseline, to estimate incident depressive symptoms or low life satisfaction, and the second longitudinal logistic regression model estimated persistent depressive symptoms or low life satisfaction. Somatic disorders and lifestyle factors were included as the main predictors and were selected based on previous literature review [4,5,7–17]. This analysis was also controlled for sociodemographic factors. Odds Ratio (OR) and 95% confidence intervals (95% CI) show the results from the logistic regressions. To decrease the probability of a Type I error, the significance level was established at *p* < 0.02. Variables significant in the univariate models were subsequently included in the multivariable model. Follow-up data were weighted accounting for attrition and mortality. All statistical procedures were conducted with StataSE 15.0 (College Station, TX, USA).

## 3. Results

### 3.1 Sample Characteristics

In total, 360 of 1932 male participants without depressive symptoms in wave 1 (24.3%) had incident depressive symptoms in wave 2 and 77 of 349 men had depressive symptoms in both waves 1 and 2 (persistent depressive symptoms). In all, 457 of 1258 male participants without low life satisfaction in Wave 1 (47.6%) had incident low life satisfaction in Wave 2, and 360 of 998 men had low life satisfaction at both Wave 1 and 2 (persistent low life satisfaction). Table 1 shows the sample characteristics of the male participants.

### 3.2 Associations with Incident Depressive Symptoms

In adjusted logistic regression analysis, not married was positively associated, and 8 to 11 years of education was negatively associated with incident depressive symptoms. In the unadjusted analysis, living with HIV and kidney disease was positively associated with incident depressive symptoms (Table 2).

### 3.3 Associations with Persistent Depressive Symptoms

Participants with 1–11 years of education had a lower probability of persistent depressive symptoms compared to those without education. Alcohol dependence and in the unadjusted analysis cardiovascular disease and general body underweight increased the odds of persistent depressive symptoms (Table 3).

### 3.4 Associations with Incident Low Life Satisfaction

In adjusted logistic regression analysis, increasing age increased the odds, while higher education, and high physical activity decreased the odds of incident low life satisfaction (Table 4).

### 3.5 Associations with Persistent Low Life Satisfaction

Increasing age and tobacco use increased the odds and high physical activity decreased the odds of persistent low life satisfaction (Table 5).

## 4. Discussion

In this first longitudinal study in Africa, we found that of the seven somatic conditions and five lifestyle factors evaluated, alcohol dependence increased the odds of persistent depressive symptoms and low physical activity, and tobacco use increased the odds of incident and/or persistent low life satisfaction among men in rural South Africa. In unadjusted analysis, living with HIV, cardiovascular disease and kidney disease were associated with incident or persistent depressive symptoms. Incident depressive symptoms in people with HIV may be explained by HIV-induced brain injury or antiretroviral medication [27]. Consistent with previous research in China [7], kidney diseases were associated with incident depressive symptoms in unadjusted analysis in this study. Patients with chronic kidney disease appear to have a higher risk of depressive illness, especially depending on the severity of the disease, which may require dialyses and cause several somatic symptoms [28].

While we found in unadjusted analysis an association between cardiovascular disease and persistent depressive symptoms among men, other studies found an association between heart disease and incident depression among both sexes [8,9]. The relationship between depression and cardiovascular disease may be bidirectional, explaining the link between cardiovascular disease and persistent depression [29]. Some other studies [10] found an association between somatic disorders (kidney disease and dyslipidemia) with low life satisfaction among men and women, while in the unadjusted analysis we only found significant associations between somatic conditions (cardiovascular disease, anemia and kidney disease) and incident low life satisfaction. Having several chronic diseases may negatively affect different body organs and increase disability, all of which can contribute to accumulation of negative emotions that lead to lower life satisfaction [30].

Furthermore, alcohol dependence was in this study among men associated with persistent depressive symptoms. Similar results were found in the four-country study [13] such that heavy drinking was associated with persistent depression. However, it is not clear if alcohol dependence was a risk factor or a consequence of persistent depression [13]. Some research [13] showed a positive association between tobacco use and depressive symptoms, while we did not find this association with depressive symptoms but with persistent low life satisfaction. More research is needed to show whether cessation of tobacco use can improve life satisfaction. Consistent with previous findings [17,18], we found that increased physical activity was associated with greater life satisfaction. Contrary to a previous review that found that higher fruit and vegetable consumption was associated with a lower chance of incident depression [16], we did not find this association. Possible reasons for this non-association could be the overall low rate of adequate consumption of fruit and vegetables (11.8%) and that anti-inflammatory effects of fruit and vegetable intake were confounded by the intake of other nutrients (not assessed in this study).

## 5. Study Limitations

Life satisfaction was only assessed with a single item and depressive symptoms were only measured with a screening questionnaire and not with a diagnostic psychiatric evaluation. Furthermore, participants who were negative for depression or low life satisfaction in wave 1 may have had depression or low life satisfaction before.

## 6. Conclusions

Of the seven somatic conditions and five lifestyle factors evaluated, those with alcohol dependence were more 4.5 times more likely to have persistent depressive symptoms, and those with low physical activity and tobacco use were more likely to have incident and/or persistent low life satisfaction among men in rural South Africa.

## Figures and Tables

**Table 1.** Sample characteristics of men 40 years and older, Agincourt, South Africa, 2014–2019.

Baseline variables	Subcategory	Sample	Depressive symptoms		Low life satisfaction	

			Incident	Persistent	Incident	Persistent

		N (%)	N (%)	N (%)	N (%)	N (%)

Sociodemographic factors						
All		2346	360 (24.3)	77 (31.3)	456 (52.4)	359 (50.9)
Age (in years)						
	40–49	398 (17.1)	61 (22.1)	9 (26.4)	68 (36.2)	42 (38.5)
	50–59	606 (26.0)	95 (23.7)	17 (26.4)	101 (39.9)	91 (43.9)
	60–69	621 (26.6)	108 (26.5)	20 (26.9)	121 (44.6)	101 (51.3)
	70–79	475 (20.4)	68 (23.8)	21 (40.5)	118 (63.4)	87 (62.2)
	80 or more	234 (10.0)	26 (25.7)	10 (41.3)	48 (71.6)	36 (66.7)
Country of birth						
	Mozambique/other	676 (29.0)	108 (24.3)	17 (30.7)	103 (47.9)	127 (48.8)
	South Africa	1653 (71.0)	251 (24.4)	59 (31.1)	353 (47.6)	232 (52.2)
Education in years						
	None	958 (41.0)	146 (26.3)	39 (42.9)	170 (55.7)	174 (55.7)
	1–7 years	819 (35.0)	132 (25.0)	28 (25.1)	182 (50.7)	120 (48.3)
	8–11	303 (13.0)	40 (18.0)	4 (12.5)	59 (41.9)	51 (50.0)
	12 or more	259 (11.1)	42 (23.7)	6 (27.6)	44 (28.1)	15 (32.9)
Marital status						
	Married/cohabiting	1603 (68.4)	237 (21.6)	50 (33.9)	327 (45.9)	239 (49.6)
	Not married	742 (31.6)	123 (30.8)	27 (27.4)	130 (51.8)	121 (53.5)
Wealth index						
	Low	957 (40.8)	146 (26.7)	38 (33.7)	147 (49.4)	173 (52.1)
	Middle	451 (19.2)	61 (20.0)	12 (27.9)	97 (48.7)	68 (51.2)
	High	938 (40.0)	153 (24.3)	27 (29.2)	213 (45.9)	119 (49.2)
Somatic conditions						
HIV positive						
	No	2043 (87.6)	302 (23.4)	70 (32.8)	391 (46.7)	321 (53.0)
	Yes	290 (12.4)	58 (31.2)	7 (19.6)	65 (54.3)	38 (38.4)
Cardiovascular disease						
	No	2225 (94.9)	350 (24.4)	66 (29.1)	434 (46.9)	339 (50.3)
	Yes	119 (5.1)	10 (20.5)	11 (54.8)	23 (64.3)	20 (63.3)
Hypertension						
	No	1033 (35.4)	170 (25.9)	32 (26.9)	192 (44.9)	165 (51.5)
	Yes	1240 (54.6)	179 (22.6)	44 (35.1)	259 (50.4)	188 (50.9)
Diabetes						
	No	1907 (89.0)	295 (23.8)	60 (31.0)	383 (48.5)	300 (50.5)
	Yes	236 (11.0)	36 (27.3)	12 (43.9)	43 (49.6)	34 (52.6)
Dyslipidemia						
	No	1059 (55.2)	169 (24.2)	37 (33.9)	230 (51.1)	164 (52.5)
	Yes	861 (44.8)	141 (26.8)	31 (32.3)	158 (45.8)	136 (50.8)
Anemia						
	No	1837 (88.7)	281 (22.8)	63 (33.8)	373 (47.2)	282 (48.8)
	Yes	235 (11.3)	32 (29.3)	6 (20.0)	46 (59.7)	32 (57.5)
Kidney disease						
	No	2246 (95.9)	342 (23.9)	70 (30.3)	436 (46.9)	347 (51.4)
	Yes	97 (4.1)	18 (33.7)	7 (45.5)	21 (65.5)	12 (40.0)
Lifestyle factors Alcohol dependence						
	No	2287 (97.6)	353 (24.5)	73 (30.4)	448 (47.5)	345 (50.5)
	Yes	57 (2.4)	7 (17.9)	4 (66.7)	9 (50.0)	14 (62.9)
Current tobacco use						
	No	1827 (78.0)	273 (23.7)	60 (31.5)	365 (51.5)	263 (48.5)
	Yes	515 (22.0)	87 (26.4)	17 (30.0)	92 (55.4)	95 (58.6)
Physical activity						
	Low	983 (42.2)	143 (26.8)	35 (32.4)	187 (55.3)	162 (55.6)
	Moderate	540 (23.2)	87 (22.8)	6 (13.6)	121 (44.9)	67 (48.5)
	High	809 (34.7)	130 (23.1)	35 (37.3)	147 (42.3)	129 (46.5)
Fruit and vegetable intake/servings/day						
	0–2	944 (40.8)	143 (23.7)	32 (30.9)	196 (50.7)	147 (53.0)
	3–4	1097 (47.4)	169 (23.8)	35 (35.4)	215 (46.0)	156 (47.8)
	≥5	274 (11.8)	46 (29.4)	9 (22.4)	44 (42.9)	45 (50.0)
Body mass index						
	Normal	1019 (47.2)	156 (23.7)	33 (31.5)	196 (47.8)	162 (49.7)
	Underweight	188 (8.7)	25 (27.1)	13 (46.8)	32 (57.0)	34 (58.0)
	Overweight	612 (28.3)	101 (23.8)	20 (31.3)	129 (45.0)	97 (51.9)
	Obesity	341 (15.8)	61 (25.5)	8 (22.6)	87 (51.0)	49 (51.0)

**Table 2.** Odds ratios for the association between somatic conditions, lifestyle factors, and incident depressive symptoms among men 40 years and older, HAALSI (2014–2019).

Baseline variables	Subcategory	Crude Odds Ratio (95% CI)	Adjusted Odds Ratio (95% CI)

Age (in years)		1.00 (0.99, 1.01)	—
Country of birth			
	Mozambique/other	1 (Reference)	—
	South Africa	1.01 (0.82, 1.25)	
Education in years			
	None	1 (Reference)	1 (Reference)
	1–7 years	0.94 (0.75, 1.17)	0.94 (0.75, 1.18)
	8–11	0.62 (0.45, 0.85)**	0.61 (0.44, 0.85)**
	12 or more	0.86 (0.63, 1.19)	0.91 (0.65, 1.28)
Marital status			
	Married/cohabiting	1 (Reference)	1 (Reference)
	Not married	1.62 (1.32, 1.99)***	1.55 (1.25, 1.92)***
Wealth index			
	Low	1 (Reference)	1 (Reference)
	Middle	0.69 (0.52, 0.90)**	0.74 (0.56, 0.98)
	High	0.88 (0.71, 1.09)	1.03 (0.81, 1.30)
Somatic conditions			
HIV positive			
	No	1 (Reference)	1 (Reference)
	Yes	1.48 (1.13, 1.95)**	1.32 (1.01, 1.74)
Cardiovascular disease			—
	No	1 (Reference)	
	Yes	0.81 (0.46, 1.44)	
Hypertension			
	No	1 (Reference)	—
	Yes	0.84 (0.69, 1.02)	
Diabetes			
	No	1 (Reference)	—
	Yes	1.21 (0.86, 1.70)	
Dyslipidemia			
	No	1 (Reference)	—
	Yes	1.15 (0.93, 1.42)	
Anemia			
	No	1 (Reference)	—
	Yes	1.40 (0.98, 1.99)	
Kidney disease			
	No	1 (Reference)	1 (Reference)
	Yes	1.64 (1.04, 2.56)*	1.58 (1.01, 2.53)
Lifestyle factors			
Alcohol dependence			
	No	1 (Reference)	—
	Yes	0.66 (0.33, 1.32)	
Current tobacco use			
	No	1 (Reference)	—
	Yes	1.15 (0.92, 1.45)	
Physical activity			
	Low	1 (Reference)	—
	Moderate	0.81 (0.63, 1.04)	
	High	0.82 (0.66, 1.03)	
Fruit and vegetable intake/servings/day			
	0–2	1 (Reference)	—
	3–4	1.01 (0.82, 1.24)	
	≥5	1.33 (0.96, 1.84)	
Body mass index			
	Normal	1 (Reference)	—
	Underweight	1.19 (0.80, 1.77)	
	Overweight	1.01 (0.80, 1.27)	
	Obesity	1.11 (0.84, 1.47)	

CI, Confidence Interval

*** *p* < 0.001

** *p* < 0.01

* *p* < 0.02.

**Table 3.** Odds ratios for the association between chronic conditions, lifestyle factors and persistent depressive symptoms among men 40 years and older, HAALSI (2014–2019).

Baseline variables	Subcategory	Crude Odds Ratio (95% CI)	Adjusted Odds Ratio (95% CI)

Age (in years)		1.02 (1.00, 1.03)	—
Country of birth			
	Mozambique/other	1 (Reference)	—
	South Africa	1.03 (0.61, 1.72)	
Education in years			
	None	1 (Reference)	1 (Reference)
	1–7 years	0.45 (0.28, 0.72)***	0.45 (0.27, 0.74)**
	8–11	0.20 (0.08, 0.52)***	0.20 (0.07, 0.54)**
	12 or more	0.52 (0.22, 1.24)	0.63 (0.26, 1.54)
Marital status			
	Married/cohabiting	1 (Reference)	—
	Not married	0.73 (0.47, 1.73)	
Wealth index			
	Low	1 (Reference)	—
	Middle	0.75 (0.41, 1.39)	
	High	0.81 (0.50, 1.30)	
Somatic conditions			
HIV positive			
	No	1 (Reference)	—
	Yes	0.51 (0.25, 1.05)	
Cardiovascular disease			
	No	1 (Reference)	1 (Reference)
	Yes	3.09 (1.47, 6.50)*	2.40 (1.02, 5.35)
Hypertension			
	No	1 (Reference)	—
	Yes	1.46 (0.95, 2.26)	
Diabetes			
	No	1 (Reference)	—
	Yes	1.71 (0.88, 3.32)	
Dyslipidemia			
	No	1 (Reference)	—
	Yes	0.93 (0.58, 1.48)	
Anemia			
	No	1 (Reference)	—
	Yes	0.48 (0.23, 1.00)	
Kidney disease			
	No	1 (Reference)	—
	Yes	2.09 (0.88, 4.96)	
Lifestyle factors			
Alcohol dependence			
	No	1 (Reference)	1 (Reference)
	Yes	4.06 (1.06, 15.58)*	4.54 (1.05, 19.66)*
Current tobacco use			
	No	1 (Reference)	—
	Yes	0.95 (0.57, 1.58)	
Physical activity			
	Low	1 (Reference)	1 (Reference)
	Moderate	0.35 (0.16, 0.74)**	0.50 (0.22, 1.10)
	High	1.25 (0.79, 1.98)	1.77 (0.99, 2.89)
Fruit and vegetable intake/servings/day			
	0–2	1 (Reference)	—
	3–4	1.21 (0.76, 1.93)	
Body mass index	≥5	0.62 (0.32, 1.22)	
	Normal	1 (Reference)	—
	Underweight	1.94 (1.00, 3.77)	
	Overweight	0.99 (0.58, 1.69)	
	Obesity	0.64 (0.31, 1.31)	

CI, Confidence Interval

*** *p* < 0.001

** *p* < 0.01

* *p* < 0.02.

**Table 4.** Odds ratios for the association between chronic conditions, lifestyle factors and incident low life satisfaction among men 40 years and older, HAALSI (2014–2019).

Baseline variables	Subcategory	Crude Odds Ratio (95% CI)	Adjusted Odds Ratio (95% CI)

Age (in years)		1.04 (1.03, 1.05)***	1.03 (1.01, 1.04)***
Country of birth			
	Mozambique/other	1 (Reference)	—
	South Africa	0.98 (0.77, 1.26)	
Education in years			
	None	1 (Reference)	1 (Reference)
	1–7 years	0.82 (0.64, 1.05)	0.91 (0.70, 1.19)
	8–11	0.57 (0.41, 0.79)***	0.76 (0.52, 1.12)
	12 or more	0.31 (0.22, 0.43)***	0.50 (0.33, 0.76)***
Marital status			
	Married/cohabiting	1 (Reference)	—
	Not married	1.27 (1.01, 1.59)	
Wealth index			
	Low	1 (Reference)	—
	Middle	0.98 (0.73, 1.30)	
	High	0.87 (0.69, 1.10)	
Somatic conditions			
HIV positive			
	No	1 (Reference)	—
	Yes	1.35 (0.99, 1.84)	
Cardiovascular disease			
	No	1 (Reference)	1 (Reference)
	Yes	2.06 (1.18, 3.61)*	1.37 (0.76, 2.45)
Hypertension			
	No	1 (Reference)	—
	Yes	1.25 (1.01, 1.54)	
Diabetes			
	No	1 (Reference)	—
	Yes	1.04 (0.72, 1.51)	
Dyslipidemia			
	No	1 (Reference)	—
	Yes	0.81 (0.65, 1.02)	
Anemia			
	No	1 (Reference)	1 (Reference)
	Yes	1.65 (1.14, 2.41)**	1.46 (0.97, 2.19)
Kidney disease			
	No	1 (Reference)	1 (Reference)
	Yes	2.18 (1.24, 3.83)**	1.61 (0.88, 2.94)
Lifestyle factors			
Alcohol dependence			
	No	1 (Reference)	—
	Yes	0.98 (0.77, 1.26)	
Current tobacco use			
	No	1 (Reference)	—
	Yes	0.85 (0.67, 1.09)	
Physical activity			
	Low	1 (Reference)	1 (Reference)
	Moderate	0.66 (0.51, 0.85)***	0.74 (0.56, 0.99)
	High	0.59 (0.46, 0.75)***	0.68 (0.52, 0.89)**
Fruit and vegetable intake/servings/day			
	0–2	1 (Reference)	—
	3–4	0.83 (0.67, 1.03)	
	≥5	0.73 (0.51, 1.04)	
Body mass index			
	Normal	1 (Reference)	—
	Underweight	1.44 (0.82, 2.23)	
	Overweight	0.89 (0.70, 1.14)	
	Obesity	1.13 (0.85, 1.52)	

CI, Confidence Interval

*** *p* < 0.001

** *p* < 0.01

* *p* < 0.02.

**Table 5.** Odds ratios for the association between chronic conditions, lifestyle factors and persistent low life satisfaction among men 40 years and older, HAALSI (2014–2019).

Baseline variables	Subcategory	Crude Odds Ratio (95% CI)	Adjusted Odds Ratio (95% CI)

Age (in years)		1.03 (1.02, 1.04)***	1.03 (1.02, 1.04)***
Country of birth			
	Mozambique/other	1 (Reference)	—
	South Africa	1.15 (0.90, 1.46)	
Education in years			
	None	1 (Reference)	1 (Reference)
	1–7 years	0.74 (0.56, 0.97)*	0.96 (0.72, 1.28)
	8–11	0.79 (0.55, 1.14)	1.09 (0.74, 1.61)
	12 or more	0.38 (0.23, 0.65)***	0.61 (0.35, 1.07)
Marital status			
	Married/cohabiting	1 (Reference)	—
	Not married	1.17 (0.91, 1.50)	
Wealth index			
	Low	1 (Reference)	—
	Middle	0.97 (0.71, 1.33)	
	High	0.89 (0.68, 1.16)	
Somatic conditions			
HIV positive			
	No	1 (Reference)	1 (Reference)
	Yes	0.55 (0.39, 0.79)***	0.71 (0.49, 1.04)
Cardiovascular disease			
	No	1 (Reference)	—
	Yes	1.68 (0.93, 3.02)	
Hypertension			
	No	1 (Reference)	—
	Yes	0.98 (0.77, 1.24)	
Diabetes			
	No	1 (Reference)	—
	Yes	1.08 (0.71, 1.64)	
Dyslipidemia			
	No	1 (Reference)	—
	Yes	0.93 (0.72, 1.21)	
Anemia			
	No	1 (Reference)	—
	Yes	1.42 (0.91, 2.22)	
Kidney disease			
	No	1 (Reference)	—
	Yes	0.63 (0.35, 1.12)	
Lifestyle factors			
Alcohol dependence			
	No	1 (Reference)	—
	Yes	1.65 (0.82, 3.32)	
Current tobacco use			
	No	1 (Reference)	1 (Reference)
	Yes	1.51 (1.14, 1.99)**	1.64 (1.23, 2.19)***
Physical activity			
	Low	1 (Reference)	1 (Reference)
	Moderate	0.75 (0.54, 1.05)	0.73 (0.52, 1.02)
	High	0.70 (0.53, 0.90)**	0.73 (0.56, 0.96)*
Fruit and vegetable intake/servings/day			
	0–2	1 (Reference)	—
	3–4	0.81 (0.63, 1.05)	
	≥5	0.88 (0.61, 1.29)	
Body mass index			
	Normal	1 (Reference)	—
	Underweight	1.40 (0.91, 2.16)	
	Overweight	1.09 (0.82, 1.45)	
	Obesity	1.05 (0.73, 1.52)	

CI, Confidence Interval

*** *p* < 0.001

** *p* < 0.01

* *p* < 0.02.
